# Structural and functional modulation of human kynurenine aminotransferase I enhances selenium-driven redox metabolism for cancer therapy

**DOI:** 10.1016/j.redox.2025.103967

**Published:** 2025-12-09

**Authors:** Arun Kumar Selvam, Renhua Sun, Tatiana Sandalova, Hugh Salter, Adnane Achour, Mikael Björnstedt

**Affiliations:** a Division of Pathology, Department of Laboratory Medicine, Karolinska Institutet, Karolinska University Hospital, Stockholm, S-141 86, Sweden; b Science for Life Laboratory, Department of Medicine Solna, Karolinska Institute, & Division of Infectious Diseases, Karolinska University Hospital, Solna, SE-171 77, Sweden

**Keywords:** Se-methylselenocysteine, Kynurenine aminotransferase 1, Transamination, β-elimination, Apoptosis, HDAC inhibition, Chromatin remodeling, Cell cycle arrest

## Abstract

Se-methylselenocysteine (MSC) is a redox-active selenium-containing amino acid with notable anticancer potential, requiring enzymatic activation for cytotoxicity. Human kynurenine aminotransferase 1 (hKYAT1) catalyzes MSC through transamination and β-elimination pathways, generating β-methylselenopyruvate and methylselenol, both of which induce oxidative stress and epigenetic modulation. To enhance MSC metabolism and its therapeutic efficacy, we performed site-directed mutagenesis targeting three critical hKYAT1 residues: Tyr101, Asp126, and Phe278. These mutants, along with wild-type hKYAT1, were expressed in hepatocellular carcinoma cell lines HepG2 and Huh7, and their impact on enzymatic activity, cytotoxic effects, apoptosis and chromatin remodeling were evaluated. Several mutations significantly enhanced MSC metabolism, with Y101H and F278A increasing both transamination and β-elimination activity, and D126L favoring β-elimination. These modifications led to a five-to 30-fold increase in MSC-induced cytotoxicity compared to wild-type hKYAT1. Additionally, mutant hKYAT1 expression altered histone deacetylase (HDAC) profiles, increased histone H4 acetylation, and activated apoptotic signaling through caspase cleavage and cytochrome *c* release. Collectively, our findings demonstrate that rational engineering of hKYAT1 can potentiate MSC metabolism and amplify its anticancer effects, offering a promising enzyme-targeting strategy for selenium-based cancer therapies.

## Highlights

• Rational engineering of hKYAT1 altered its catalytic balance between transamination and β-elimination.
• Mutants Y101H and F278A enhanced dual activity towards Se-methylselenocysteine (MSC).
• D126L selectively favoured β-elimination and induced redox stress–driven cytotoxicity.
• Enhanced MSC metabolism by hKYAT1 mutants led to apoptosis and histone acetylation in cancer cells.
• Findings support hKYAT1 as a tuneable metabolic node for selenium-based cancer therapy.

## Graphical abstract

**Figure:**
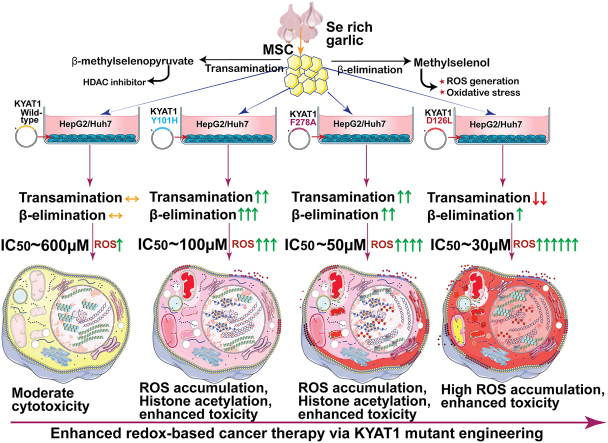

## 1 Introduction

Se-methylselenocysteine (MSC) is a well-characterized selenium compound known for its bioavailability, low cytotoxicity, favorable pharmacokinetic properties and potent anticancer activity upon enzymatic activation [1]. MSC remains inert in its native form and requires enzymatic processing by kynurenine aminotransferase 1 (KYAT1, KAT1) also known as cysteine-S-conjugate beta-lyase 1 (CCBL1), glutamine transaminase K (GTK), to exert its biological effects. KYAT1 catalyzes MSC via two pathways: β-elimination to yield methylselenol (MS), a reactive oxygen species (ROS) generator, and transamination to form β-methylselenopyruvate (MSP), a known histone deacetylase (HDAC) inhibitor [2,3]. Both metabolites contribute to cancer-selective cytotoxicity through oxidative stress, chromatin remodeling, and apoptosis induction [4,5]. These dual, mechanistically distinct pathways underscore MSC's selective cytotoxicity in malignant cells while minimizing systemic toxicity to benign cells.

Human KYAT1 (hKYAT1) is a pyridoxal 5′-phosphate (PLP)-dependent, promiscuous aminotransferase that catalyzes reactions involving large neutral, aromatic, and sulfur/selenium-containing amino acids including kynurenine [6,7]. The enzyme catalyzes both transamination and β-elimination reactions via a ping-pong bi–bi mechanism, where both pathways share a common quinonoid intermediate that dictates the metabolic outcome [6,8,9]. While transamination is generally the dominant reaction, substrates with nucleophilic leaving groups can shift this balance in favor of β-elimination [10]. The functional KYAT1 enzyme exists as a dimer, with each monomer comprising a flexible N-terminal arm (residues 1–17), a small domain (residues 18–43 and 302–421), and a large domain (residues 44–301) [11]. The active site resides at the dimer interface and incorporates residues from all three structural regions. Key catalytic residues include Asp126, Trp18, Tyr101, and Phe278, which are essential for substrate binding, PLP stabilization, and transition state stabilization. Prior mutagenesis studies have underscored the importance of Trp18, Phe125, and His279 for substrate recognition and enzyme catalysis [7]. Our earlier work demonstrated that overexpression of wild-type hKYAT1 in hepatocellular carcinoma (HCC) cell lines enhances MSC metabolism and selectively induces cytotoxicity, even at low MSC concentrations [12]. Building upon these findings, we hypothesized that the strategic mutagenesis of active-site residues could enhance hKYAT1's catalytic efficiency toward MSC, thereby amplifying its anticancer effects. Specifically, we targeted residues Tyr101 (gatekeeper), Asp126 (helix α1 stabilizer), and Phe278 (substrate ring interactor), based on their structural importance and proximity to the PLP-binding site. This study examines the effects of these targeted mutations on MSC metabolism, cytotoxicity, HDAC modulation, and apoptotic signaling in HCC cells. By coupling structure-guided mutagenesis with functional analysis, we aim to improve the therapeutic potential of MSC via enzyme engineering of KYAT1.

## 2 Materials and methods

### 2.1 Chemicals and reagents

Se-methylselenocysteine (Se-Methylselenocysteine hydrochloride, MSC), α-Keto-γ-methylthiobutyric acid sodium salt (KMB), Dimethyl-2-oxoglutarate (α-KG), 2-Amino-2-methyl-1,3-propanediol, Pyridoxal 5′-phosphate hydrate (PLP), Potassium dihydrogen phosphate, Di-potassium hydrogen phosphate, Ethylenediaminetetraacetic acid (EDTA), Sodium arsenate dibasic heptahydrate, L-Tryptophan (L-Trp), l-Kynurenine (L-Kyn), l-Histidine (L-His), L-Phenyl alanine (L-Phe), dl-Tyrosine (dL-Tyr), Phenylpyruvic acid (PPA), 2-keto-butyric acid (KBA), l-Leucine (L-Leu), l-Alanine (L-Ala), l-Glutamine (L-Gln), dl-Methionine (dL-Met), l-Cystine (L-Cys), l-Asparagine (L-Asn), l-Aspartic acid (L-Asp), Glycine (Gly), Proline (Pro), Selenomethionine (SeMet), Phenylmethanesulfonyl fluoride (PMSF), RIPA buffer, Protease inhibitor cocktail mix, N–N-Dimethyl formamide, and Sodium hydroxide were purchased from Sigma‒Aldrich (St. Louis, MO, USA). NADPH was obtained from Acros Organics (Geel, Belgium). Lipofectamine 3000 was procured from Invitrogen (Camarillo, CA, USA). The pEGFP-N1 plasmid (Clontech, Takara Bio Inc, Mountain View, CA, USA) was kindly provided by Dr. Gilbert Lauter and Dr. Peter Swoboda, Department for Biosciences and Nutrition, Karolinska Institutet, Stockholm, Sweden. PageRuler Plus Prestained protein ladder was purchased from Thermo Fischer Scientific (Rockford, IL, USA). Mammalian TrxR1 was obtained from Sigma‒Aldrich (Cas no: 9074-14-0, Product number T9698, Darmstadt, Germany). The CellTiter-Glo® 2.0 Cell Viability Assay Kit was purchased from Promega (Madison, WI, USA).

### 2.2 Cell culture and growth conditions

HepG2 cells were acquired from ATCC (Wesel, Germany) and Huh7 cells were generously provided by Dr. Camilla Pramfalk (Division of Clinical Chemistry, Karolinska University Hospital Huddinge, Sweden). Both cell lines were maintained in Eagle's Minimum Essential Medium (EMEM; ATCC) supplemented with 10 % heat-inactivated fetal bovine serum (FBS; Gibco, Paisley, UK). Cultures were incubated at 37 °C in a humidified atmosphere containing 5 % CO_2_. No antibiotics were added to the medium. To ensure experimental integrity, both cell lines were regularly screened for mycoplasma contamination using the MycoAlert™ Mycoplasma Detection Kit (Lonza, Boston, MA, USA). Cell density and viability were routinely assessed using a TC20™ automated cell counter (Bio-Rad, Portland, ME, USA) to maintain consistency across experimental replicates.

### 2.3 Cloning, expression, and purification of recombinant hKYAT1 and mutated variants

Key residues in the hKYAT1 were selected for site-directed mutagenesis based on *in silico* modeling and structural analysis using available crystal structures from the Protein Data Bank (PDB). Mutations targeted residues involved in substrate stabilization, catalytic activity, and active site conformation. Wild-type hKYAT1 was cloned and expressed as described previously [12]. Constructs encoding the desired hKYAT1 variants for mammalian cell transfection and recombinant protein expression were generated using standard molecular biology techniques.

### 2.4 Transient transfection of hKYAT1 and mutant constructs

HepG2 and Huh7 cells were seeded at a density of 1 × 10^6^ cells per well in 6-well plates and cultured for 24 h. Cells were transfected with 2 μg of plasmid DNA (wild-type or mutant hKYAT1) using Lipofectamine™ 3000 (Invitrogen) following the manufacturer's protocol. Cells were harvested 48 h post-transfection for downstream assays, including enzymatic activity, and Western blotting.

### 2.5 Cell viability assay

Cell viability was assessed using the CellTiter-Glo® 2.0 Luminescent Cell Viability Assay (Promega), following the manufacturer's protocol. Briefly, cells were harvested 20 h post-transfection (wild-type and mutants), and seeded at a density of 400 (HepG2) or 300 (Huh7) cells/mm^2^ in 96-well plates. After 24 h, cells were treated with varying concentrations of MSC for an additional 72 h. Luminescence was measured using a CLARIOSTAR® (BMG Labtech, Ortenberg, Germany), and viability was expressed as a percentage relative to untreated controls.

### 2.6 Recombinant protein production

Recombinant protein production involved transforming BL21 *E. coli* competent cells with plasmid DNA, followed by heat shock and overnight incubation on ampicillin LB agar plates. A single colony was then used to prepare an overnight culture. For protein expression, the overnight culture was diluted into fresh LB medium, grown to an optical density (OD) at 600 nm of 0.6, cooled, and induced with 0.5 mM IPTG at 16 °C for 20 h. Cells were harvested by centrifugation, then lysed via sonication in lysis buffer. Further purification was performed using a His Trap™ Nickel column (Merck), followed by sequential elution with 100 mM and 500 mM imidazole. Eluted protein was further purified by size-exclusion chromatography to achieve up to 95 % purity, with the KYAT1 dimer protein having an estimated apparent molecular weight of 113 kDa.

**Nuclear and cytoplasmic extraction:** Nuclear and cytoplasmic proteins were extracted using the EpiQuik™ Nuclear extraction kit I (#OP-0002, EPIGENTEK, Farmingdale, NY, USA) according to the manufacturer's instruction. Briefly, cells were fractionated to isolate nuclear and cytoplasmic components under optimized conditions to preserve protein integrity.

**Whole-cell lysate preparation:** For whole-cell lysates, cells were lysed on ice for 30 min in RIPA buffer containing 1 mM PMSF and 1 % protease inhibitor cocktail mix. The lysed cells were subjected to sonication at 4 °C for 30 s using 1-s pulses. Proteins were extracted by centrifugation at 13,000 rpm for 10 min at 4 °C. The supernatant containing soluble proteins was carefully collected for further analysis.

**Protein quantification:** Protein concentrations for all cell extracts were determined using the Pierce™ Bicinchoninic Acid (BCA) protein assay kit (Thermo Fisher Scientific, Waltham, MA, USA), following the manufacturer's protocol.

### 2.7 Transamination assays using l-phenylalanine

Transamination activity for l-phenylalanine (L-Phe) was performed as previously described [13]. In brief, a 50 μL reaction mixture containing 20 mM L-Phe, 5 mM α-keto-γ-methiolbutyrate (KMB), 100 mM ammediol-HCl buffer (pH 9.0), and either 200 ng of purified human KYAT1 (hKYAT1) or 20 μg of whole cell lysate protein was incubated at 37 °C. Reactions with 0–100 mM L-Phe were used to determine Michaelis–Menten kinetics. After 30 min, reactions were terminated with 150 μL of 1 M NaOH, and phenylpyruvate-enol (PPA-enol) formation was quantified by absorbance at 320 nm (*ε* = 16,000 M^−1^cm^−1^) using a PowerWave HT spectrophotometer (BioTek) in UV-transparent flat-bottom plates (Costar). Reactions without enzyme served as blanks.

### 2.8 Transamination assays using MSC, SeMet, and other amino acids

Transamination with other amino acids (L-Leu, L-Ala, L-Cys, L-Gln, DL-Met, Gly, Pro, SeMet, and MSC) followed a modified protocol [14]. Reactions (50 μL) were prepared in 200 mM potassium phosphate EDTA buffer (pH 7.4) containing 5 mM MSC or SeMet, and 0.6 mM phenylpyruvic acid. Mixtures were pre-incubated at 37 °C for 5 min before addition of enzyme (200 ng purified hKYAT1 or 20 μg of lysate). Kinetic assays used 0–10 mM MSC or 0–15 mM SeMet. Reactions were stopped after 10 min by adding 150 μL of 1 N NaOH, and phenylpyruvate consumption was measured at 320 nm.

### 2.9 Transamination assays using l-tryptophan, dl-tyrosine, and l-histidine

Transamination of l-tryptophan (L-Trp), dl-tyrosine (DL-Tyr), and l-histidine (L-His) was evaluated using a modified method [15]. Briefly, a 100 μL reaction mixture was prepared, containing 3 mM of the respective amino acid (L-Trp, dL-Tyr, or L-His), 5 mM α-keto-γ-methylthiobutyrate (KMB), 70 μM PLP, and 330 mM ammediol-HCl buffer (pH 9.6). Either 20 μg of whole-cell lysate or 200 ng of recombinant protein was added to the reaction, which was then incubated at 37 °C for 30 min. The reaction was terminated by adding 7 μL of 50 % trichloroacetic acid (TCA), followed by vortexed, and centrifugation to remove precipitated proteins. For product detection, 50 μL of the reaction supernatant was mixed with 250 μL of 1 M arsenate-borate reagent (pH 6.0), vortexed, and incubated at room temperature for 30 min. Absorbance was measured using a spectrophotometer (PowerWave HT, BioTek, Winooski, VT, USA) at 292 nm for L-His (Imidazolepyruvate, *ε* = 11,300 M^−1^cm^−1^), 310 nm for DL-Tyr (*p*-Hydroxyphenylpyruvate, *ε* = 10,700 M^−1^cm^−1^), and 330 nm for L-Trp (Indolepyruvate, *ε* = 10,800 M^−1^cm^−1^). A blank control was prepared by adding KMB immediately before TCA addition.

### 2.10 Transamination assays using l-kynurenine

Transamination of l-kynurenine (L-Kyn) to kynurenic acid (L-Kyna) using α-ketoisocaproate (KBA) as the acceptor was carried out in 50 μL reactions containing 3 mM L-Kyn, 5 mM KBA, 200 μM PLP, and 300 mM ammediol-HCl buffer (pH 9.6), with 400 ng hKYAT1 or 40 μg lysate. 0–5 mM L-Kyn was used for kinetic analysis. Reactions were incubated at 37 °C for 6 h and stopped with 3.5 μL of 50 % TCA. After centrifugation, 180 μL of 500 mM phosphate buffer (pH 7.5) was added to 20 μL of supernatant, and absorbance was measured at 330 nm (*ε* = 8850 M^−1^cm^−1^) [16]. KBA was added just before TCA for blank controls.

For all transamination kinetic measurements (enzyme concentration and incubation time) were optimized in pilot experiments to ensure <10–15 % substrate consumption and strictly linear product formation over the quench interval. Under these conditions, product formed at a single fixed time point reflects the initial velocity (v_0_), which was used to calculate k_obs_ and fit Michaelis-Menten parameters.

### 2.11 β-Elimination assays using MSC and SeMet

β-elimination activity toward MSC and SeMet was evaluated as previously described [13,17]. Reactions (100 μL) included 100 mM potassium phosphate buffer (pH 7.4), 5 mM MSC or SeMet, 100 μM each of α-ketoglutarate and KMB, 10 μM PLP, 0.5 μg recombinant mammalian thioredoxin reductase 1 (TrxR1), 400 μM NADPH, and either 200 ng hKYAT1 or 20 μg of cell lysate. 0–10 mM MSC or 0–15 mM SeMet was used for kinetics. After 5 min pre-incubation (excluding TrxR1 and NADPH), reactions were initiated by adding TrxR1 and NADPH. NADPH consumption was monitored at 340 nm every 30 s using a PowerWave HT spectrophotometer. Reactions without TrxR1 served as blanks. The molar extinction coefficient for NADPH was 6220 M^−1^cm^−1^. TrxR1 was provided in excess to ensure accurate kinetic evaluation.

**Supplementary Fig. 1:**
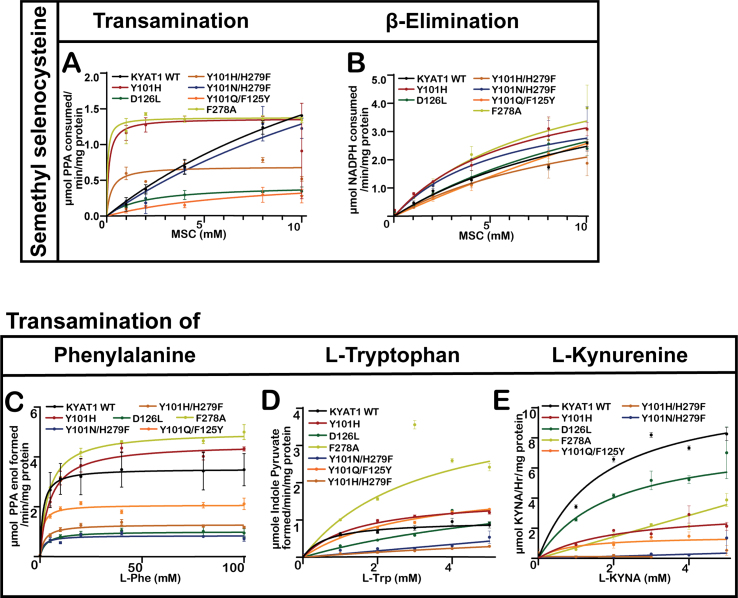
Michaelis–Menten enzyme kinetics of KYAT1 wild-type and mutant enzymes in transamination and β-elimination assays. **(A, B)** Michaelis–Menten curves of recombinant KYAT1 wild-type (WT) and mutants were gexlnerated using MSC as substrate to evaluate **(A)** transamination activity via PPA-enol formation and **(B)** β-elimination activity by measuring NADPH consumption. **(C**–**E) Transamination MM-kinetics were assessed for**l-phenylalanine **(C),**l-tryptophan **(D),** and l-kynurenine **(E)** as amino acid substrates. All reactions were conducted using purified recombinant proteins. Data represent mean ± SEM from three independent experiments. Kinetic parameters (K_m_ and V_max_) were calculated using nonlinear regression in GraphPad Prism.

For L-Phe, L-Trp, L-Kyn, MSC, and SeMet, steady-state kinetic parameters (K_m_, k_cat_, k_cat_/K_m_) were obtained from Michaelis–Menten fits across multiple substrate concentrations (Supplementary Figs. 1 and 3). For conditions in which Michaelis-Menten fitting did not converge, results are reported as ND (not determined). For other amino acids (e.g., Ala, Gly, Cys, Met, Gln, and Pro), only single-concentration endpoint assays were conducted and used to compare WT and KYAT1 mutant variants.

### 2.12 Western blot analysis

Transfected cells were lysed using RIPA buffer supplemented with a protease inhibitor cocktail (Sigma–Aldrich) and PMSF (1 mM). Protein concentrations were determined using a BCA assay (Thermo Fisher Scientific). Equal amounts of protein (20 μg for whole cell lysates/cytoplasmic extract and 30 μg for nuclear extract) were separated on SDS–PAGE gels (Bio-Rad, Portland, ME, USA) and transferred onto PVDF membranes (Bio-Rad, Portland, ME, USA). Membranes were blocked with 5 % non-fat milk in 1XTBST for 2 h at room temperature and probed with primary antibodies in 1 % BSA in 1XTBST (Supplementary Table 1) overnight at 4 °C. After washing, membranes were incubated with HRP-conjugated secondary antibodies (polyclonal swine anti-rabbit immunoglobulins/HRP (P0399) and/or polyclonal goat anti-mouse immunoglobulins/HRP (P0447)) (DAKO, Glostrup, Denmark), diluted 1:10,000 in 5 % milk. After secondary antibody incubation, membranes were washed three times with 1 × TBST, and bands were visualized using the Odyssey Fc Imaging System (LI-COR®, Nebraska, USA). Densitometric analysis was performed using Odyssey Image software (LI-COR Biosciences®, Nebraska, USA).

### 2.13 SDS-PAGE and native PAGE

SDS-PAGE was performed for the preliminary assessment of protein purity and for molecular weight determination prior to Native PAGE analysis. Native PAGE was subsequently performed to analyze the proteins under non-denaturing conditions, thereby preserving their native conformation and quaternary structure. Electrophoresis was conducted using Mini-PROTEAN® TGX™ Precast Gels (Bio-Rad, Portland, ME, USA), which are SDS-free. For Native PAGE, the running buffer followed the standard Tris/glycine formulation but was prepared without SDS. The Native PAGE was run at a constant voltage of 180 V for 40 min at room temperature, whereas the SDS-PAGE gel was run at 200 V for 30 min.

### 2.14 Measurement of cellular oxidative stress

Intracellular oxidative stress were assessed in Huh7 cells transfected with empty vector, wild-type hKYAT1 or its mutants F278A and D126L. Cells were seeded in 6-well plates and transfected using Lipofectamine™ 3000. After 24 h of transfection, cells were treated with MSC at the indicated concentrations (250 and 500 μM) for an additional 24 h. Following treatment, cells were incubated with 10 μM CM-H_2_DCFDA (Thermo Fisher Scientific) in serum-free medium for 30 min at 37 °C in the dark. After staining, cells were harvested, washed with PBS, and analyzed using a CytoFLEX flow cytometer (Beckman Coulter) at 495/535 nm (ex/em). A total of 20,000 events were collected per sample. Antimycin A (10 μM) and N-acetyl cysteine (300 μM) were used as positive and negative controls, respectively. The percentage of CM-H_2_DCFDA -positive cells was determined by applying a fixed fluorescence threshold gated with live cell population in the FITC (CM-H_2_DCFDA) channel and normalized to untreated controls.

### 2.15 Determination of GSH/GSSG levels

Intracellular reduced (GSH) and oxidized (GSSG) glutathione were quantified using the Glutathione Assay Kit (Sigma-Aldrich, Cat.No.38185) following the manufacturer's protocol with minor modifications. Briefly, Huh7 cells transfected with hKYAT1 (empty vector, wild-type or mutants F278A and D126L) were treated with MSC (250 or 500 μM, 24 h), lysed by three freeze-thaw cycles in 10 mM HCl, and deproteinized with 5 % sulfosalicylic acid (SSA). Supernatants were analyzed for total and oxidized glutathione, and GSH levels were calculated as GSH = Total-2xGSSG. The GSH/GSSG ratio was used to assess redox balance. *Tert*-butyl hydroperoxide (tBHP; 200 μM, 30 min), hydrogen peroxide (H_2_O_2_; 20 μM, 30 min), and buthionine sulfoximine (BSO; 200 μM, 24 h) were used as positive controls to verify assay sensitivity to oxidative stress and glutathione synthesis inhibition.

### 2.16 Extracellular H_2_O_2_ quantification

Extracellular hydrogen peroxide was quantified using the Amplex Red Hydrogen Peroxide/Peroxidase Assay Kit (Thermo Fisher Scientific, Cat.No: A22188) according to the manufacturer's protocol. Briefly, Huh7 cells transfected with hKYAT1 (empty vector, wild-type or mutants F278A and D126L) were seeded in black, clear-bottom 96-well plates and pre-equilibrated in phenol red-free, serum-free medium. Cells were treated with MSC (250 or 500 μM) in the presence or absence of aminooxyacetic acid (AOAA, 1 mM; 1 h preincubation) for 1 h at 37 °C. Rotenone (200 nM), antimycin A (10 μM), and tBHP (200 μM) were included as positive controls for mitochondrial and chemical oxidative stress, respectively. Immediately before measurement, Amplex Red (50 μM final) and horseradish peroxidase (HRP; 0.1 U/mL) were added to each well. Fluorescence (Ex 560 nm/Em 590 nm) was recorded every 5 min for 75 min using a CLARIOSTAR® microplate reader (BMG Labtech, Ortenberg, Germany). The slope of the linear region (5–35 min; R^2^ ≥ 0.99) was used to calculate the rate of H_2_O_2_ generation (RFU·min^−1^). Rates were background-corrected, normalized to viable cell number (CellTiter-Glo assay), and expressed as the percentage of extracellular H_2_O_2_ generation relative to untreated control (set to 100 %).

### 2.17 BODIPY 581/591C11 lipid peroxidation assays

Lipid peroxidation was quantified using BODIPY™ 581/591C11 (Thermo Fisher Scientific, Cat.No. D3861). Briefly, Huh7 cells transfected with hKYAT1 (empty vector, wild-type or mutants F278A and D126L) were seeded in black 96-well plates (clear bottoms) and treated with MSC (250 and 500 μM, 12 h). Following treatment, cells were incubation with the probe (5 μM) in phenol-red-free, serum-free HBSS buffer for 30 min at 37 °C. Fluorescence was measured using a CLARIOSTAR® microplate reader (BMG Labtech, Ortenberg, Germany) at excitation/emission wavelengths of 490/540 nm (oxidized) and 570/600 nm (reduced). Blank-corrected values were expressed as the Oxidation Index (Green/Red) and normalized to the mean of ferrostatin-1-treated wells (1 μM; Fer-1 = 1) to confirm lipid-ROS specificity. *Tert*-butyl hydroperoxide (tBHP, 200 μM, 30 min) and antimycin A (10 μM, 30 min) served as positive controls for general and mitochondrial oxidative stress, respectively.

### 2.18 Mitochondrial superoxide measurements

Mitochondrial superoxide levels were quantified using MitoSox™ Red mitochondrial superoxide indicator (Thermo Fisher Scientific, Cat.No. M36008). Briefly, Huh7 cells were transfected with an empty vector, KYAT1 wild-type, or mutants F278A and D126L. At 24 h post-transfection, cells were treated with MSC (250 or 500 μM) for 12 h. Antimycin A (10 μM, 30 min) and tBHP (200 μM, 30 min) were used as positive controls for mitochondrial and general oxidative stress, respectively. Following treatment, cells were incubated with MitoSOX™ Red (5 μM, 20 min, 37 °C, protected from light), washed twice with HBSS, and stained with IR Dead Cell Staining Kit (1 μg/mL, Thermo Fisher Scientific, Cat.No. L34976) to exclude non-viable cells. Flow cytometry was performed on a CytoFLEX flow cytometer (Beckman Coulter), and data were analyzed in FlowJo (BD Biosciences). The percentage of MitoSOX-positive cells was determined by applying a fixed fluorescence threshold gated on the live cell population in the PE (MitoSox) channel.

### 2.19 Statistical analysis

Data were analyzed using GraphPad Prism 10.1.2 software (GraphPad Software, San Diego, CA, USA). Results are presented as mean ± standard deviation (SD) of at least three independent experiments. Statistical comparisons were made using one-way analysis of variance (ANOVA) with a 95 % confidence interval, followed by Dunnett's multiple comparison test for group comparisons. For IC50 values, nonlinear regression slopes were fitted to data from independent experiments (n ≥ 3). Differences between groups were analyzed using one-way ANOVA followed by Tukey's multiple comparison test. A *p*-value <0.05 was considered statistically significant.

## 3 Results

**Fig. 1:**
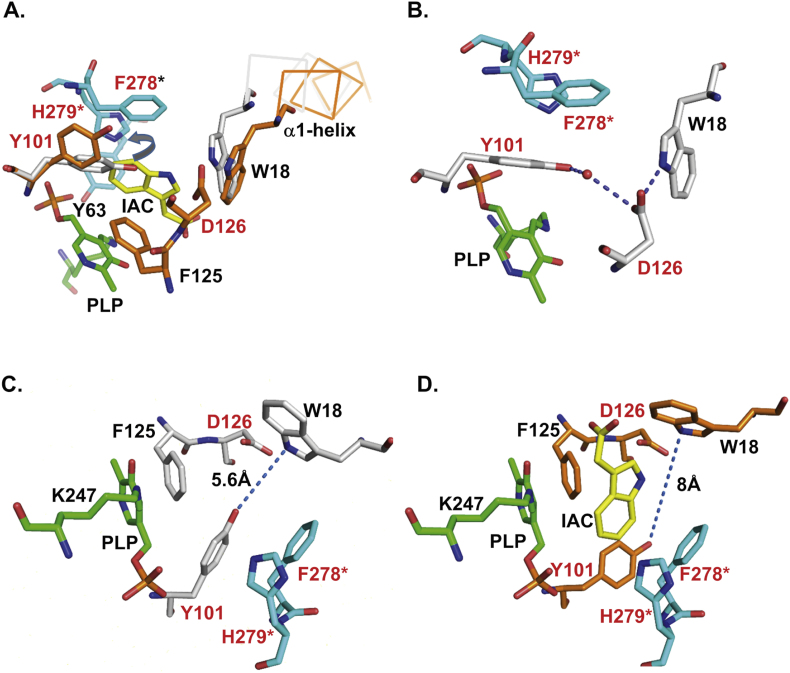
**Ligand binding induces the conformational changes of several residues in the KYAT1 active site.** Superposition of the crystal structure of KYAT1 in its free state (3FVS.pdb, shown in gray) and in complex with the inhibitor IAC (yellow) (3FVU.pdb, shown in orange). Active site residues from the second subunit of KYAT1 are depicted in light blue and marked with an asterisk (*). Residue K247 and the covalently bound cofactor PLP are shown in green. Mutated residues analyzed in this study: Y101, D126, and F278 are labeled in red. The orientation of all panels was manually adjusted to provide optimal visualization of the displayed residues. The figures were created using PyMol [18]. **(A)** Comparison of the crystal structures of free KYAT1 (3FVS.pdb) and complex KYAT1 (3FVU.pdb) demonstrates that ligand binding induces conformational changes in helix α1 and residue Y101. **(B)** Residue D126 connects the two mobile residues, Y 101 and W18, from helix α1 in KYAT1. **(C)** In the free KYAT1 state, Y101 is positioned close to W18. **(D)** The rotation of Y101 creates space for ligand binding, while residues F278* and H279* act as a rigid wall in the active site. Each panel was manually oriented to optimize the visualization of the residues displayed.

***Choice of mutations*.** The functional KYAT1 enzyme exists as a dimer. The substrate binding site of hKYAT1 consists of several aromatic residues from both monomers. These include Trp18, Tyr101, and Phe125 from one monomer, and Tyr63*, His279*, and Phe278* (* indicates residues from the other subunit) from the second monomer (Fig. 1A). Notably, the best hKYAT1 substrates, such as L-Phe, L-Trp, kynurenine, and L-Gln possess planar regions that are well-suited for binding near the aromatic residues of the enzyme. The crystal structure of the dimeric hKYAT1 revealed that Tyr101 resides in one monomer, while Phe278* resides in the other, located parallel to the substrate binding site. Additional aromatic contact is provided by Phe278*, whose aromatic ring is oriented almost perpendicular to the ligand ring. The mutations of Trp18, Phe125, and His279, previously described in our earlier publication, underscore the importance of these interactions. Interestingly, Phe278* is the sole residue in hKYAT1 that resides in the disallowed region of the Ramachandran plot, suggesting that its unique conformation plays a pivotal role in substrate recognition and catalysis. This observation raises important questions about the factors governing the differing substrate specificities among hKYAT1 isozyme.

Most active site residues in hKYAT1 remain static during substrate binding. However, two notable exceptions are helix α1 and the side chain of Tyr101, which undergo conformational changes. Helix α1 covers the active center from the solvent, and its lateral the movement is likely necessary for substrate penetration into the binding site (Fig. 1A). In the ligand-free state, the Tyr101 side chain rotates from its original position to open the gate for the incoming substrate (Fig. 1A). Another key residue, Asp126 which is located between Trp18 and Tyr101, may influence the movement of helix α1 as well as the conformation of the Tyr101 side chain (Fig. 1B). In the ligand-free hKYAT1 structure (PDB code 1W7L), residue Asp126 forms a hydrogen bond with the hydroxyl group of Tyr101, either directly or via a water molecule (Fig. 1B). However, in other ligand-free KYAT1 structures (PDB 4WLH), this water molecule is absent, and instead, a direct hydrogen bond is formed between Asp126 and the N1*ε* atom of Trp18. This interaction remains intact in complexes with IAC (PDB 3FVU) and L-Phe (PDB 1W7M). The importance of these hydrogen bonds was further investigated using the D126L mutant. Our results suggest that Asp126 stabilizes the N-terminus of the α1-helix, preventing it from excessive movement, even during the relatively large displacement of the C-terminus of the α1-helix during substrate binding [11,19].

The hydroxyl group of Tyr101 shifts away by 3.5 Å from Asp126, forming a hydrophobic pocket for the ligand. Movements of Tyr101 and helix α1 occur in opposite directions (Fig. 1C and D). This dynamic behavior allows the active site to adapt to the size of the substrate. The distance between the hydroxyl group of Tyr101 and Nε of Trp18 varies from 8 Å in the complex with indole-3-acetic acid (IAC, PDB ID: 3FUV), to 6.3 Å in the complex with L-Phe, 5 Å in the complex with glycerol or Tris and only 4.7 Å in the substrate-free form. To investigate the role of Tyr101 further, we mutated this residue to His101, Phe101, Met101, Asn101 and Gln101 to maintain the possibility that it forms hydrogen bonds. Here, we focus on the roles of hKYAT1 residues Tyr101, Asp126, and Phe278, which are critical for substrate entry and stabilization within the binding pocket.

### 3.1 hKYAT1 mutants enhance MSC-induced cytotoxicity in HCC cell lines

**Fig. 2:**
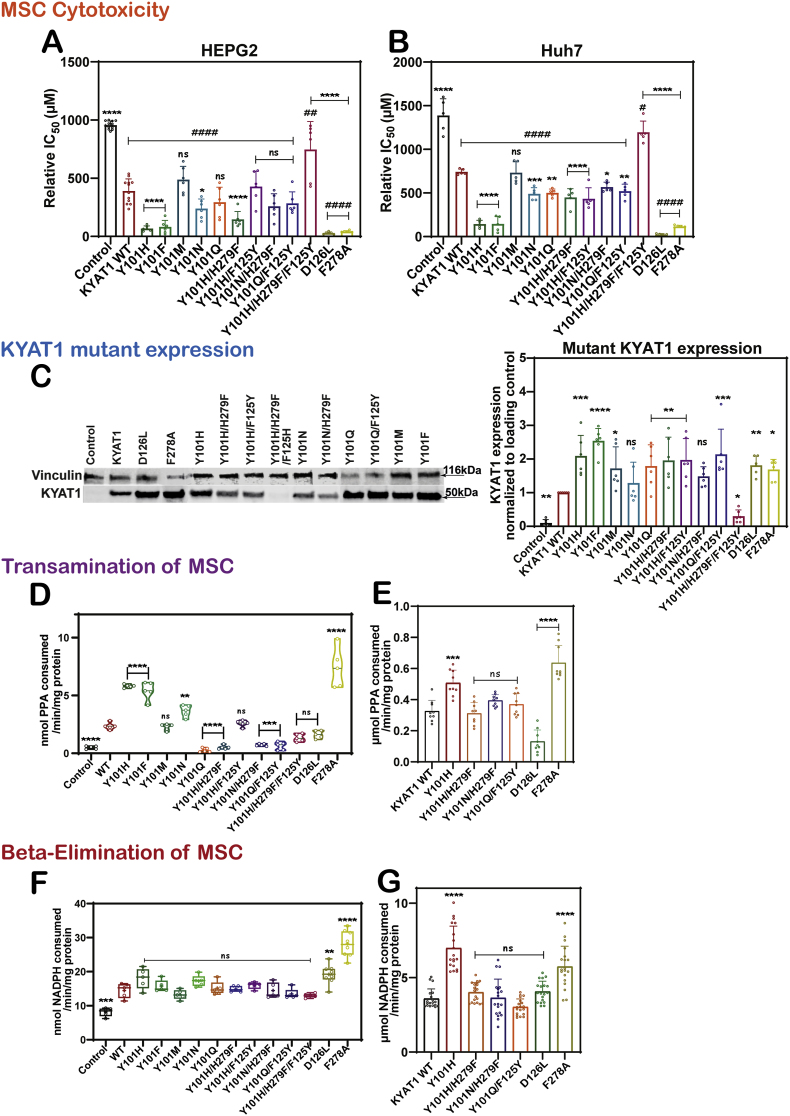
**MSC cytotoxicity in cells overexpressing wild-type or mutanted hKYAT1 variants (A)** HepG2 cells (n = 5), **(B)** Huh7 cells (n = 5). **(C) h**KYAT1 wild-type and mutant expressions levels were quantified using Western blot (n = 3). Transamination and β-elimination of MSC by hKYAT1 wild-type and mutants: Transamination done with **(D)** Cell lysate (n = 6), **(E)** Recombinant protein (n = 6). β-elimination assay done with **(F)** Cell lysate (n = 6), **(G)** Recombinant protein (n = 6). (**A-G**) The graph represents mean ± SD, statistical analysis performed with one-way ANOVA with 95 % confidence interval followed by Tukey's multiple comparison test (ns = not significant, *p < 0.05, **p < 0.01, ***p < 0.001, and ****p < 0.0001 compared with wild-type KYAT1).

To investigate whether the targeted KYAT1 mutations can enhance MSC metabolism, wild-type and each mutant KYAT1 variants were overexpressed in hepatocellular carcinoma (HCC) cell lines (HepG2 and Huh7). Compared to empty vector controls, wild-type KYAT1 increased MSC-induced cytotoxicity approximately 2-fold, consistent with our previous findings. Among all mutants, D126L exhibited the strongest enhancement, leading to a 30-fold (IC_50_ = 25 μM) and 13-fold (IC_50_ = 30 μM) increase in MSC cytotoxicity in Huh7 and HepG2 cells, respectively (Fig. 2A and B). The F278A mutation conferred strong cytotoxic effects, with a 6-fold (IC_50_ = 117 μM) increase in Huh7 and 9-fold (IC_50_ = 46 μM) in HepG2. This phenotype is consistent with the enzyme's relatively high catalytic efficiency toward MSC; however, the pronounced cytotoxicity suggests that conformational changes resulting from the loss of the bulky aromatic side chain may further facilitate substrate access or product release in the cellular context (Fig. 2).

Y101H emerged as the most potent among all the Tyr101 mutants, increasing MSC-induced cytotoxicity 5-fold (IC_50_ = 173 μM) in Huh7 and 7-fold (IC_50_ = 87 μM) in HepG2, corresponding to its dual enhancement of transamination and β-elimination activity. The Y101F mutant displayed comparable effects, consistent with increased transamination but not β-elimination activity. In contrast, mutants like Y101 M and Y101H/H279F/F125Y did not significantly affect cytotoxicity, reflecting their minimal enzymatic activity. Other mutants like Y101 N, Y101Q, Y101H/H279F, Y101H/F125Y, and Y101Q/F125Y displayed variable cytotoxic responses, with the stronger effects generally observed in Huh7 cells (Fig. 2A and B).

Western blot analysis confirmed the successful expression of most mutants, with levels approximately 2-fold higher than wild-type KYAT1 (Fig. 2C). However, differences in MSC sensitivity did not correlate with protein abundance, indicating that catalytic efficiency, rather than expression level, is the primary determinant of cytotoxic potency.

### 3.2 Gatekeeper residue Y101 modulates MSC metabolism via transamination and β-elimination

To evaluate the functional impact of mutations at the Y101 gatekeeper residue, we assessed both transamination and β-elimination activity in whole-cell lysates and recombinant KYAT1 proteins using MSC as the substrate (Fig. 2D–G). Substitution of Y101 with histidine (Y101H) markedly enhanced both catalytic pathways. Transamination activity increased approximately 2-fold in whole-cell lysates and 1.5-fold in recombinant proteins compared to wild-type KYAT1. Kinetic analysis revealed a substantial increase in catalytic efficiency (*K*cat/*K*m = 46,370 s^−1^ mM^−1^) compared to the wild-type (754 s^−1^ mM^−1^), driven by improved substrate affinity (*K*m = 0.1 vs. 24.3 mM) and high turnover (*K*cat = 4526 vs. 18,360 s^−1^) (Supplementary Fig. 1A, Table 1). A similar 2-fold enhancement in transamination activity was observed with the aromatic substitution mutant Y101F, suggesting that maintaining aromaticity at this position supports MSC catalysis. In contrast, amidic substitutions produced divergent effects. Y101 N retained elevated activity, while Y101Q abolished transamination, underscoring subtle differences in side-chain chemistry. Y101 M showed no elevated activity over wild-type (Fig. 2D). Next, the effect of double and triple mutations combining Y101 substitutions with F125Y or H279F were evaluated. The double mutants Y101H/H279F and Y101Q/F125Y exhibited transamination activities comparable to wild-type, while Y101H/F125Y showed moderate enhancement in whole-cell lysates. Notably, the triple mutant Y101H/H279F/F125Y failed to surpass wild-type activity, suggesting that combinatorial substitutions of these residues do not synergize effectively with MSC (Fig. 2D and E, Supplementary Fig. 1A, Table 1).

**Table 1:** Michaelis-Menten kinetics parameter for transamination and β-elimination of the different mutants for different amino acid substrates.

Amino acid	Mutant KYAT1	*K*m (mM)	*K*cat (s^−1^)	*K*cat/*K*m (s^−1^ mM^−1^)
**L-Phe**	WT	1.6 ± 0.7	13301 ± 592	8313 ± 3656
	Y101H	4.8 ± 0.6	17026 ± 383	3547 ± 451
	Y101H/H279F	2.3 ± 0.5	4937 ± 144	2147 ± 471
	Y101 N/H279F	1.8 ± 0.8	3154 ± 163	1752 ± 784
	Y101Q/F125Y	1.2 ± 0.4	7842 ± 211	6535 ± 2185
	D126L	3.1 ± 0.8	3925 ± 163	1266 ± 331
	F278A	4.9 ± 0.5	119138 ± 353	24300 ± 2500
**L-Trp**	WT	0.8 ± 0.2	3861 ± 256	4826 ± 1248
	Y101H	1.4 ± 0.1	5881 ± 195	4201 ± 331
	Y101H/H279F	13.7 ± 9.9	3981 ± 2255	291 ± 267
	Y101 N/H279F	ND	ND	ND
	Y101Q/F125Y	3.2 ± 0.5	7869 ± 548	2459 ± 421
	D126L	11.7 ± 12.5	13530 ± 10862	1156 ± 1545^a^
	F278A	2.2 ± 1.4	15720 ± 4140	7145 ± 4921
**L-Kyn**	WT	1.7 ± 0.7	2147 ± 187	1263 ± 532
	Y101H	3.7 ± 0.9	6988 ± 1756	1889 ± 661
	Y101H/H279F	0.14 ± 0.2	255 ± 427	1821 ± 4009^a^
	Y101 N/H279F	ND	ND	ND
	Y101Q/F125Y	1.2 ± 0.3	2234 ± 478	1862 ± 613
	D126L	11.0 ± 2.2	20661 ± 4190	1878 ± 535
	F278A	ND	ND	ND
**MSC**	WT	24.3 ± 7.8	18360 ± 4425	756 ± 303
	Y101H	0.1 ± 0.1	4526 ± 434	45260 ± 45468^a^
	Y101H/H279F	0.2 ± 0.2	2522 ± 201	12610 ± 12650^a^
	Y101 N/H279F	26.8 ± 23.1	18461 ± 12244	689 ± 749^a^
	Y101Q/F125Y	5.1 ± 3.4	1639 ± 500	321 ± 236
	D126L	1.6 ± 0.5	1543 ± 125	964 ± 311
	F278A	0.1 ± 0.1	5249 ± 141	52490 ± 52509^a^
**SeMet**	WT	17.9 ± 6.3	2668 ± 592	149 ± 62
	Y101H	29.6 ± 18.9	5503 ± 2548	186 ± 147
	Y101H/H279F	7.2 ± 4.0	1051 ± 271	146 ± 89
	Y101 N/H279F	4.2 ± 3.4	236 ± 75	56 ± 49
	Y101Q/F125Y	3.8 ± 1.3	2213 ± 274	582 ± 212
	D126L	0.1 ± 0.3	343 ± 43	3430 ± 10299^a^
	F278A	141.8 ± 392.4	9399 ± 23916	66 ± 249^a^
**Beta-elimination activity**				
**MSC**	WT	11.6 ± 4.4	18946 ± 4402	1633 ± 727
	Y101H	7.0 ± 1.7	20315 ± 2529	2902 ± 792
	Y101H/H279F	8.4 ± 3.6	13257 ± 3089	1578 ± 770
	Y101 N/H279F	8.2 ± 3.1	21359 ± 4428	2605 ± 1123
	Y101Q/F125Y	48.7 ± 25.7	56793 ± 25643	1166 ± 810
	D126L	11.9 ± 4.2	21273 ± 4646	1788 ± 742
	F278A	9.7 ± 4.9	26104 ± 7635	2691 ± 1571
**SeMet**	WT	0.5 ± 0.3	18142 ± 1641	36284 ± 22016^a^
	Y101H	0.3 ± 0.3	14190 ± 1542	47300 ± 47578^a^
	Y101H/H279F	0.6 ± 0.8	10420 ± 2429	17367 ± 23507^a^
	Y101 N/H279F	ND	ND	ND
	Y101Q/F125Y	ND	ND	ND
	D126L	0.3 ± 0.4	5260 ± 769	17533 ± 23518^a^
	F278A	0.03 ± 0.3	9643 ± 1218	321433 ± 3214590^a^

ND = not determined due to inability to obtain reliable kinetic fits. Steady-state kinetic parameters (*Km, Kcat, Kcat/Km*) are reported for L-Phe, L-Trp, L-Kyn, MSC and SeMet, where full Michaelis-Menten analyses across multiple substrate concentration were performed (See Supplementary Figs. 1 and 3).

a Large SD values in Kcat/Km reflect error propagation from nonlinear regression, particularly for variants with low activity or shallow substrate saturation curves; these values should be interpreted cautiously as ratio-derived parameters.

For β-elimination, only Y101H demonstrated a significant increase (1.5-fold) in recombinant proteins, with a catalytic efficiency (*K*cat/Km = 2901 s^−1^ mM^−1^) nearly double that of wild-type (1631 s^−1^ mM^−1^) (Fig. 2F and G, Supplementary Fig. 1B, Table 1). Other Y101-based double mutants (Y101H/H279F, Y101 N/H279F, Y101Q/F125Y) retained β-elimination activity near baseline levels, indicating limited additive effects. Together, these data establish residue Y101 as a critical determinant of MSC catalysis. Specific substitutions, particularly Y101H and Y101F, reconfigure the substrate entry gate and enhance enzymatic turnover, leading to improved MSC metabolism and cytotoxicity.

### 3.3 F278A enhances dual catalytic activity, while D126L selectively promotes β-elimination of MSC

To dissect the role of key active-site residues in MSC metabolism, we evaluated the effects of F278A and D126L mutations on KYAT1 enzymatic activity and cytotoxic function. The F278A mutation significantly enhanced MSC transamination activity, showing a 12-fold increase in mock-transfected lysates and a 3-fold increase in wild-type overexpressing lysates (Fig. 2D). The recombinant F278A retained a 2-fold increase in activity over recombinant wild-type KYAT1 (Fig. 2E). Kinetic analysis confirmed a marked improvement in catalytic efficiency (*K*cat/*K*m = 41,277 vs 754 s^−1^ mM^−1^ for wild-type), driven by increased turnover and affinity (Supplementary Fig. 1A, Table 1). Similarly, the β-elimination activity was enhanced, increasing 3.5-fold in mock lysates, and 2-fold in wild-type lysates and recombinant proteins (Fig. 2F and G). Altogether, our results, indicate that the F278A mutant favors a dual enhancement of both catalytic pathways. In contrast, the D126L mutation shifted the KYAT1 activity selectively toward β-elimination. The transamination activity was abolished in recombinant protein but remained comparable to wild-type in whole-cell lysates (Fig. 2D and E), suggesting a possible compensatory effect in the cellular environment. The β-elimination activity was significantly elevated in whole-cell lysates (Fig. 2F), while recombinant D126L showed no change compared to wild-type (Fig. 2G). Kinetic analysis showed modest catalytic efficiency (*K*cat/*K*m = 952), (Supplementary Fig. 1A, Table 1, Supplementary Table 2), underscoring that this mutation may affect the conformational transitions required for MSC β-cleavage without enhancing intrinsic turnover. Altogether, these results highlight distinct functional roles for F278 and D126 in substrate processing. While the F278A mutation broadly boosts MSC metabolism through improved catalytic kinetics, D126L selectively promotes β-elimination, most likely by altering α1-helix dynamics and thus the overall conformation of the active site.

### 3.4 MSC treatment reshapes HDAC expression and histone acetylation in KYAT1-overexpressing cells

**Fig. 3:**
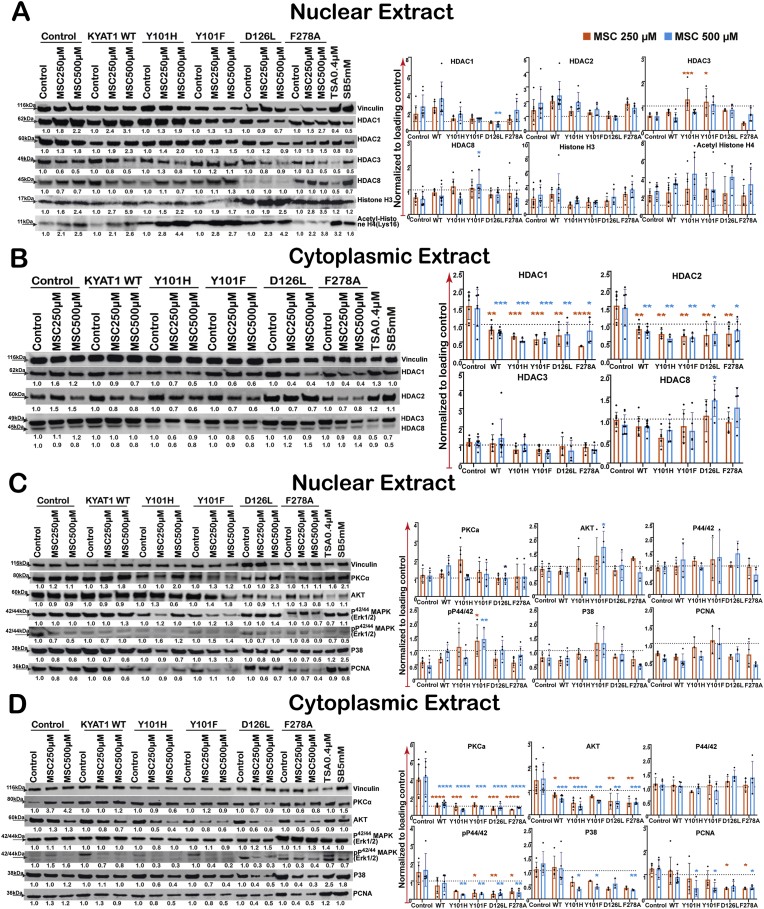
**MSC treatment modifies the expression of class I histone deacetylases and increases the acetylation of histone levels in hKYAT1 wild-type and mutant over-expressing cells.** Western blot was done with (**A**) Nuclear extract (n = 3–5) (**B**) Cytoplasmic extract (n = 3–5). MSC treatment increases the cell cycle arrest and anti-proliferative effect by regulating the gene expression responsible for the proliferation in both hKYAT1 wild-type and mutant over-expressed HepG2 cells. Western blot was performed with (**C**) Nuclear extract (n = 3–6), (**D**) Cytoplasmic extract (n = 3–5). (**A-D**) (ns = not significant, *p < 0.05, **p < 0.01, ***p < 0.001, and ****p < 0.0001; Green denotes significance compared with control lysate treated with 250 μM of MSC, Blue denotes significance compared with control lysate treated with 500 μM of MSC).

MSC treatment induced distinct changes in class I histone deacetylase (HDAC) expression and histone acetylation, depending on both MSC concentration and the specific KYAT1 variants that are expressed. In control cells, MSC treatment increased nuclear HDAC1 and HDAC2 expression in a dose-dependent manner, while HDAC3 and HDAC8 levels declined (Fig. 3A). Concurrently, acetylation of histone H4 at lysine 16 (H4K16Ac) and total histone H3 levels increased, indicating chromatin decondensation and potential transcriptional activation. Overexpression of wild-type hKYAT1 further amplified nuclear HDAC1 and HDAC2 expression under MSC treatment, while suppressing HDAC3. In contrast, the HDAC8 levels remained unchanged. This shift was accompanied by increased nuclear H4K16 acetylation and histone H3 accumulation, suggesting that MSC bioconversion via hKYAT1 enhances epigenetic remodeling.

Among all mutants, Y101H and Y101F induced the most pronounced changes, markedly increasing nuclear HDAC3, and HDAC8 levels compared to wild-type hKYAT1. These effects were especially strong at 250 μM MSC, indicating a higher catalytic turnover and metabolite generation by these mutants. Conversely, the D126L mutant did not alter the expression levels of nuclear HDAC1–3, nor HDAC8 levels, but increased H4K16Ac and histone H3, suggesting no HDAC activity modulation due to low transamination and high β-elimination function. (Fig. 3A). The F278A mutant behaved similarly to wild-type, increasing nuclear HDAC1 and HDAC2 while downregulating HDAC3, exerting no effect on HDAC8. Notably, all mutants except Y101F displayed higher H4K16 acetylation levels compared to wild-type at 500 μM MSC, reinforcing the link between enhanced MSC metabolism and chromatin remodeling. (Fig. 3A).

Cytoplasmic HDAC levels were also affected by the MSC treatment, which led to reduced cytoplasmic HDAC1 and HDAC2 in all mutant hKYAT1-overexpressing cells. However, the D126L mutation uniquely caused a dose-dependent accumulation of cytoplasmic HDAC8. This distinct pattern may reflect the bias of this mutation toward β-elimination, generating metabolite profiles that differentially influence HDAC dynamics. (Fig. 3B). Together, these findings demonstrate that MSC metabolism via KYAT1 and through the Y101H, F278A and D126L mutants, modulates nuclear and cytoplasmic HDAC expression levels in a mutation-specific manner. These changes very likely contribute to the epigenetic and apoptotic outcomes observed in MSC-treated liver cancer cells.

### 3.5 KYAT1-mediated MSC metabolism disrupts PKCα, AKT, and MAPK signaling in a mutation-specific manner

We examined the effects of MSC treatment on key signaling pathways, including PKCα, AKT, and MAPKs, in HepG2 cells overexpressing wild-type and mutant KYAT1. Subcellular fractionation revealed that MSC metabolism significantly altered the localization and abundance of these signaling proteins in a mutation-specific manner (Fig. 3C and D). PKCα nuclear accumulation increased in a dose-dependent manner in cells overexpressing wild-type hKYAT1, as well as Y101H, and Y101F, suggesting an enhanced nuclear signaling activation. In contrast, other mutants, including D126L and F278A, did not exhibit significant nuclear PKCα enrichment, indicating limited impact on this axis (Fig. 3C). AKT displayed an inverse pattern since MSC treatment led to increased cytoplasmic AKT in control cells, while the overexpression of hKYAT1 (wild-type and most mutants) reduced the cytoplasmic AKT levels in a dose-dependent fashion. Interestingly, the Y101F mutant uniquely exhibited increased nuclear AKT levels following MSC treatment, implying a distinct impact of this mutation on survival signaling via AKT translocation (Fig. 3C and D). Furthermore, MAPK signaling was broadly suppressed by MSC. Cytoplasmic p38 MAPK and phosphorylated p44/42 MAPK (*p*-Erk1/2) were downregulated across most hKYAT1-expressing cells, including wild-type and all tested mutants. However, total cytoplasmic Erk1/2 levels remained unchanged, except in F278A and D126L-expressing cells, which showed induction at 500 μM MSC. In contrast, nuclear *p*-Erk1/2 was elevated in Y101F-expressing cells, suggesting mutant-specific enhancement of nuclear MAPK activity despite reduced cytoplasmic levels. (Fig. 3C and D). Nuclear p38 MAPK expression decreased with MSC in all conditions except for Y101F, which showed increased nuclear p38, implying altered stress kinase signaling. Furthermore, the cell survival and proliferation markers PCNA were strongly downregulated in cytoplasmic fractions of cells expressing Y101H, Y101F, D126L, or F278A, correlating with enhanced MSC cytotoxicity in these mutants (Fig. 3C and D). Collectively, these results demonstrate that MSC metabolism via hKYAT1 reprograms significantly intracellular signaling networks. The Y101H, D126L, and F278A mutants exerted the strongest inhibitory effects on pro-survival signaling, through coordinated suppression of AKT, PKCα, and MAPK activity, reinforcing their roles in sensitizing cells to MSC-induced apoptosis**.**

### 3.6 KYAT1 mutant-mediated MSC metabolism activates mitochondrial apoptosis pathways

**Fig. 4:**
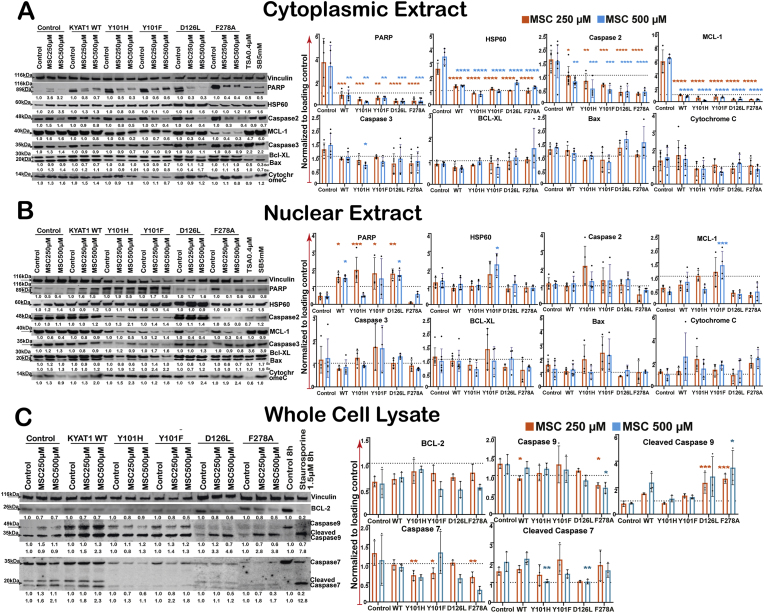
**MSC treatments increase the apoptotic events both in hKYAT1 wild-type and mutant over-expressed cells.** (**A**) Nuclear extract (n = 3), (**B**) Cytoplasmic extract (n = 3–5), (**C**) whole cell lysate (n = 3–5), (**A-C**) (ns = not significant, *p < 0.05, **p < 0.01, ***p < 0.001, and ****p < 0.0001; Green denotes significance compared with control lysate treated with 250 μM of MSC, Blue denotes significance compared with control lysate treated with 500 μM of MSC).

To evaluate the pro-apoptotic effects of MSC metabolism, we examined the expression and cleavage status of key apoptotic regulators in HepG2 cells overexpressing wild-type or mutant KYAT1 after MSC treatment (Fig. 4A–C). MSC exposure led to robust activation of the intrinsic mitochondrial apoptotic pathway. In cells expressing wild-type hKYAT1, MSC increased the levels of cleaved caspase-9 and cytochrome *c* release, compared to control cells. Cleaved caspase-9 levels were most elevated in F278A and D126L-expressing cells treated with 500 μM MSC (Fig. 4A–C). Cleaved caspase-7 and caspase-9 levels were most elevated in Y101F-expressing cells treated with MSC, along with strong upregulation of PARP, HSP60, MCL-1, caspase-3 and Bax. Cytochrome *c* release was also significantly enhanced in these mutants, confirming mitochondrial outer membrane permeabilization as a key event in MSC-induced cell death. Notably, F278A induced moderate but consistent activation of the same apoptotic markers (Fig. 4A and B).

MSC also reduced expression of anti-apoptotic proteins including MCL-1, Bcl-XL, and Bcl-2, particularly in cells overexpressing D126L and F278A. In contrast, HSP60 levels remained largely unaffected except for Y101F. PARP cleavage, another hallmark of apoptosis, was clearly detectable in all MSC-treated KYAT1-overexpressing cells, with the strongest signal in the D126L mutant. (Fig. 4A and B). A corresponding decline in cell proliferation markers, including PCNA and phosphorylated Erk1/2 (Pp44/42 MAPK), was observed, especially in Y101H-, D126L- and, F278A-expressing cells, further supporting a shift from survival to apoptotic signaling (Fig. 3D). Furthermore, MSC treatment also partially reduced BCL-2 expression and induced the activation of cleaved Caspase 7 and Caspase 9, both essential components of the apoptotic cascade in whole-cell lysates in a dose-dependent manner (Fig. 4C).

### 3.7 KYAT1 expression enhances MSC-induced oxidative stress and redox imbalance in Huh7 cells

**Fig. 5:**
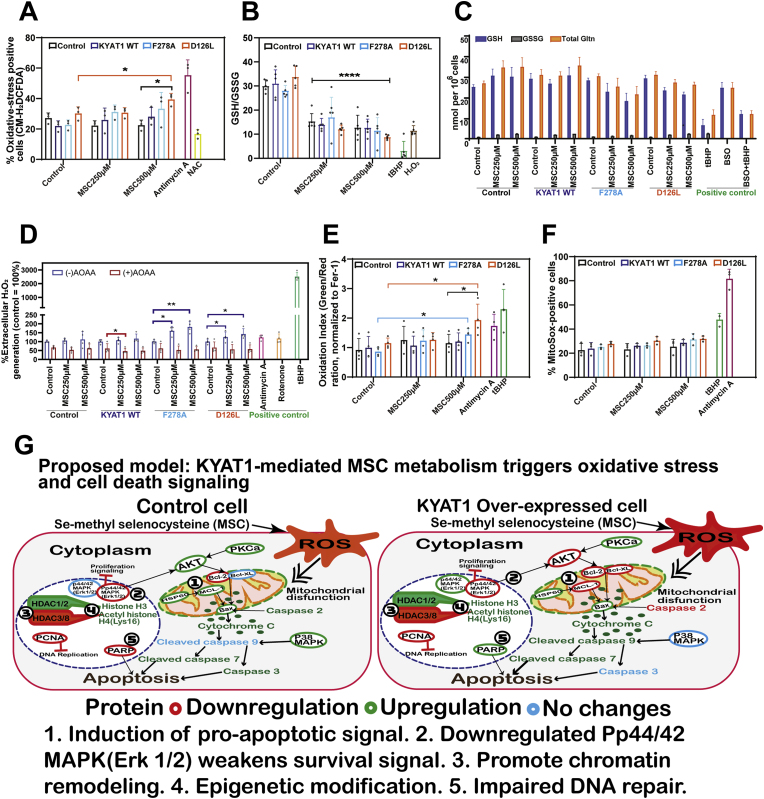
**Integrated analysis of oxidative and redox responses induced by Se-methyl selenocysteine (MSC) in KYAT1-transfected Huh7 cells. (A)** Global intracellular oxidative stress measured by the probe CM-H_2_DCFDA following MSC treatment (250 & 500 μM, 24 h) in Huh7 cells transfected with empty vector, KYAT1 wild-type (WT), F278A, or D126L. Antimycin A (10 μM) and N-acetyl-l-cysteine (NAC, 300 μM) were used as positive and negative controls, respectively. **(B, C)** Intracellular reduced (GSH) and oxidized (GSSG) glutathione levels and Cellular redox balance determined by the GSH/GSSG ratio. MSC (250 & 500 μM) and KYAT1 WT/mutant expression reduced the ratio, indicating glutathione oxidation and redox imbalance. Data were normalized to cell number; H_2_O_2_ (20 μM) and tBHP (200 μM) with or without BSO (200 μM, 24h) served as oxidative and depletion controls respectively. **(D)** Extracellular H_2_O_2_ production quantified by the Amplex Red/HRP assay. Transfected (WT or mutant) Huh7 cells were treated with MSC (250 or 500 μM, 1 h) with or without AOAA (1 mM; 1 h pre-incubation). Rotenone (200 nM, 30 min), antimycin A (10 μM, 30 min), and tBHP (200 μM, 30 min) were used as positive controls. Values represent % extracellular H_2_O_2_ relative to untreated control. MSC treatment elevated H_2_O_2_ generation, which was enhanced by KYAT1 WT/mutants and attenuated by AOAA. **(E)** Lipid peroxidation detected by BODIPY™ 581/591C11 oxidation index (Green/Red) and normalized to ferrostatin-1 (Fer-1 = 1). **(F)** Mitochondrial superoxide formation assessed by MitoSOX™ Red. MSC caused a dose-dependent rise in MitoSOX-positive cells. Antimycin A (10 μM) and tBHP (200 μM) served as positive controls. **(G)** Proposed model summarizing the integrated redox and signaling responses triggered by KYAT1-mediated MSC metabolism, including ROS generation, HDAC modulation, chromatin remodeling, and caspase-dependent apoptosis. Data represent mean ± SD from n ≥ 3 independent experiments. Statistical analysis was performed using one-way ANOVA (95 % confidence interval) followed by Dunnett's multiple comparison test (*ns* = not significant, *p < 0.05, **p < 0.01, ***p < 0.001, and ****p < 0.0001 compared with wild-type KYAT1).

MSC treatment increased the fraction of oxidative-stress-positive cells, with D126L showing the strongest response and F278A and WT exhibiting intermediate increases at 500 μM (Fig. 5A). Cellular thiol redox shifted toward oxidation, as evidenced by a decrease in the GSH/GSSG ratios and overall depletion of reduced GSH (Fig. 5B and C). Extracellular H_2_O_2_ levels, measured using the Amplex Red/HRP assay, rose in KYAT1-expressing cells and were partially suppressed by AOAA, consistent with enzyme-dependent redox cycling; F278A displayed consistently high and often maximal rates (Fig. 5D). AOAA inhibited H_2_O_2_ release by approximately 30–60 % (Supplementary Fig. 4A). Lipid peroxidation, assessed by the BODIPY-C11 green/red ratio, increased in a dose-dependent manner, with the order D126L > F278A ≥ WT (Fig. 5E). Mitochondrial superoxide (MitoSOX) was marginally elevated in D126L, followed by F278A and WT compared to control cells (Fig. 5F).

Together, these complementary assays demonstrate that KYAT1 catalysis toward β-elimination (D126L) markedly amplifies MSC-driven oxidative stress, spanning thiol depletion, extracellular H_2_O_2_ release, lipid peroxidation, and mitochondrial O_2_, whereas mutants with dual-pathway activity (such as F278A) generate robust but comparatively lower redox signals. The integration of redox signaling with HDAC modulation and apoptotic markers supports the mechanistic model summarized in Fig. 5G, wherein enhanced MSC metabolism via engineered hKYAT1 promotes ROS accumulation, HDAC suppression, chromatin remodeling, and caspase-dependent apoptosis. These findings indicate that hKYAT1 mutations not only intensify MSC metabolic flux but also potentiate apoptosis, reinforcing their potential as therapeutic enhancers in selenium-based redox cancer strategies.

### 3.8 Mutations in hKYAT1 alter substrate specificity across diverse amino acids

**Fig. 6:**
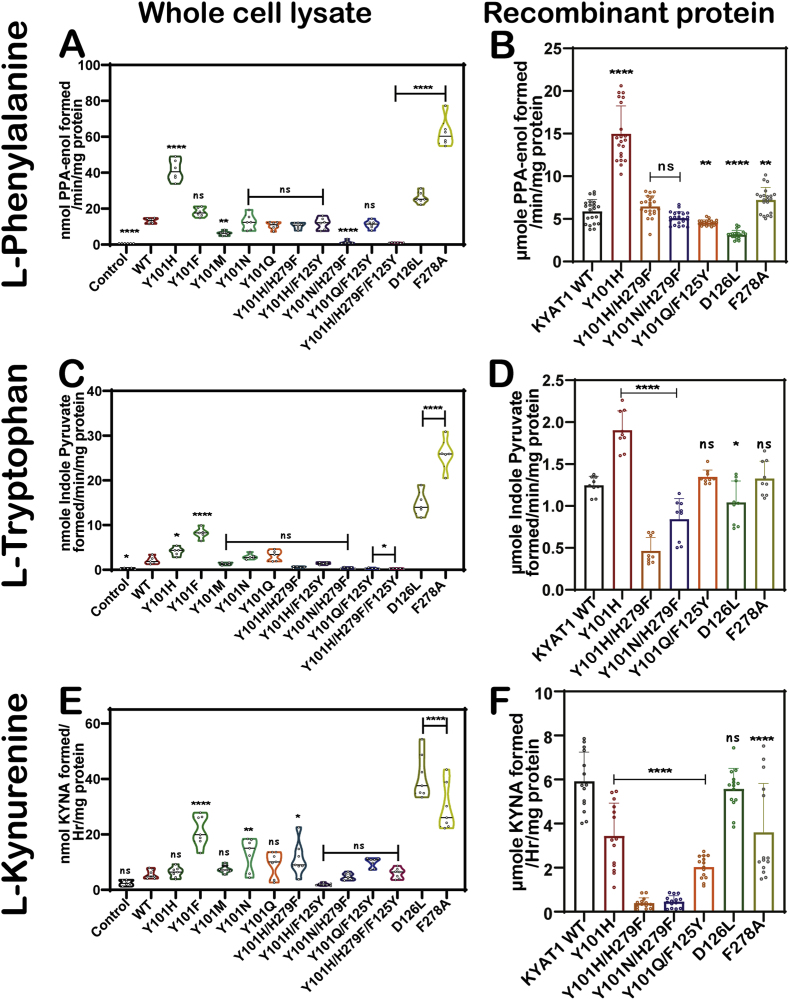
**Transamination activity of hKYAT1 with Aromatic Amino acids and****l****-Kynurenine.** Transamination activity assays were done with L-Phe. L-Trp and L-Kyn using (**A, C, E**) cell lysate (n = 6), (**B, D, F**) recombinant protein (n = 6). (**A-F**) The graph represents mean ± SD, statistical analysis performed with one-way ANOVA with 95 % confidence interval followed by Tukey's multiple comparison test (ns = not significant, *p < 0.05, **p < 0.01, ***p < 0.001, and ****p < 0.0001 compared with wild-type KYAT1) (n < 6).

**Supplementary Fig. 2:**
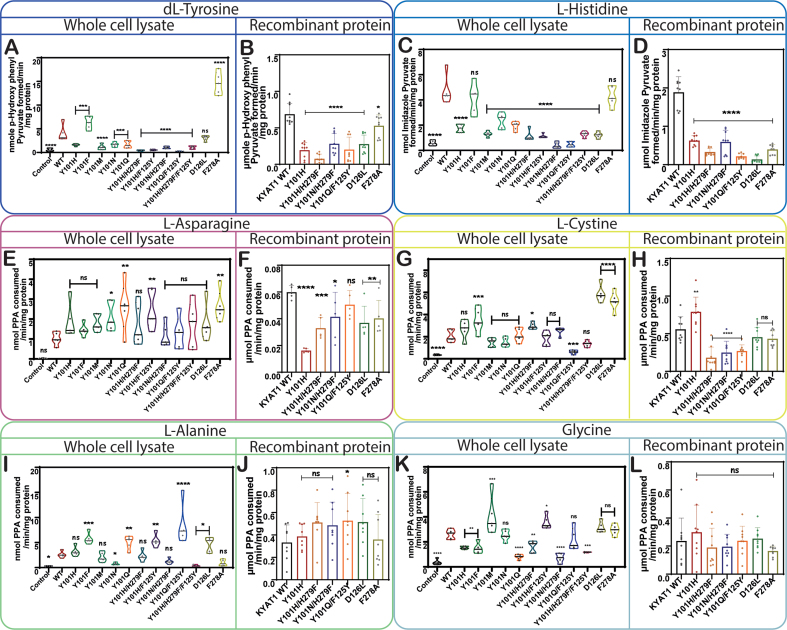
Effect of hKYAT1 wild-type and mutation with the different amino acid substrates. Transamination activity assays were done with substrates: dL-Tyr, L-His, L-Asp, L-Cyss, L-Ala and Gly (**A, C, E, G, I & K**) cell lysate, (**B, D, F, H, J & L**) recombinant protein. (**A-L**) The graph represents mean ± SD, statistical analysis performed with one-way ANOVA with 95 % confidence interval followed by Tukey's multiple comparison test (*ns* = not significant, *p < 0.05, **p < 0.01, ***p < 0.001, and ****p < 0.0001 compared with wild-type KYAT1) (n = 5).

**Supplementary Fig. 3:**
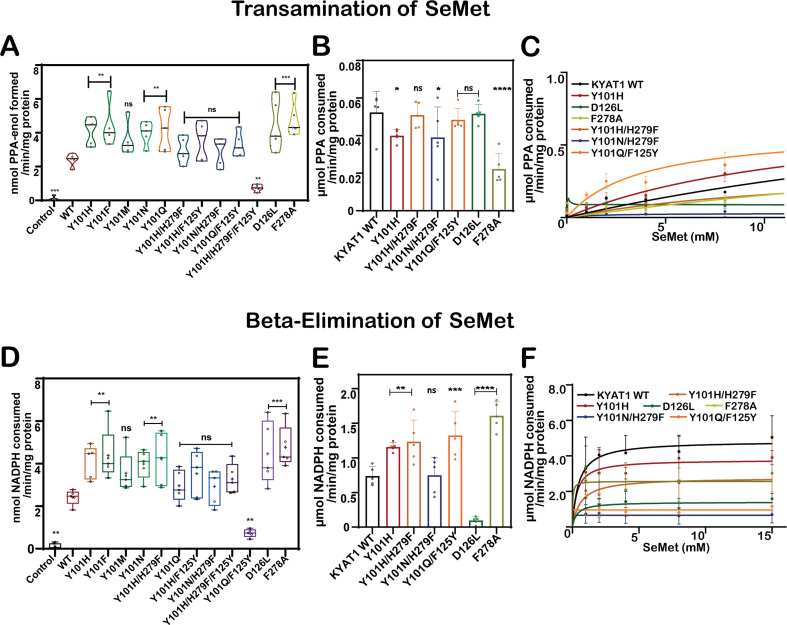
Transamination and β-elimination of SeMet by hKYAT1 wild-type and mutants: Transamination assay done with (**A**) cell lysate, (**B**) recombinant protein, (**C**) Michaelis Menten kinetics. β-elimination for SeMet was done with (**D**) cell lysate, (**E**) recombinant protein, (**F**) Michaelis Menten kinetics. (**A, B, D, E**) The graph represents mean ± SD, statistical analysis performed with one-way ANOVA with 95 % confidence interval followed by Tukey's multiple comparison test (*ns* = not significant, *p < 0.05, **p < 0.01, ***p < 0.001, and ****p < 0.0001 compared with wild-type KYAT1) (n = 6). (**C, F**) Data are represented as mean ± SEM (n = 3).

To assess whether the mutations introduced in hKYAT1 influence the substrate scope beyond MSC, we also analyzed the transamination and β-elimination activities of wild-type and mutant hKYAT1 using a panel of amino acid substrates, including l-tryptophan (L-Trp), dl-tyrosine (DL-Tyr), l-histidine (L-His), l-kynurenine (L-Kyn), l-glutamine (L-Gln), l-alanine (L-Ala), glycine, l-leucine, l-cystine, and Se-methionine (SeMet). All reactions were performed using either whole-cell lysates or a selection of purified recombinant proteins and monitored by substrate-specific spectrophotometric readouts (Fig. 6A–F, Supplementary Figs. 1–3). As expected, aromatic and related biomolecules substrates such as L-Phe, L-Trp, DL-Tyr, and L-Kyn were efficiently transaminated by wild-type hKYAT1. Among the tested mutants, F278A consistently exhibited enhanced activity toward all three substrates and L-Kyn, with significantly increased formation of respective keto acids in cell lysates (Fig. 6A–F, Supplementary Fig. 2A and B). Kinetic analysis revealed a substantial increase in catalytic efficiency of these substrates except for L-Kyn by Y101H and F278A mutants (Supplementary Fig. 1C–E, Table 1). For certain variants (e.g., F278A with L-Kyn), measurable activity was observed in endpoint assays; however, Michaelis-Menten fitting did not converge to reliable parameters, and these are therefore reported as ND in Table 1. Whereas the Y101H mutant reduced the enzyme activity towards dL-Tyr and L-His in both whole-cell lysates and recombinant proteins compared to wild-type (Supplementary Fig. 2A–D). Notably, D126L showed minimal transamination activity for these substrates, consistent with its enzymatic bias toward β-elimination.

**Fig. 7:**
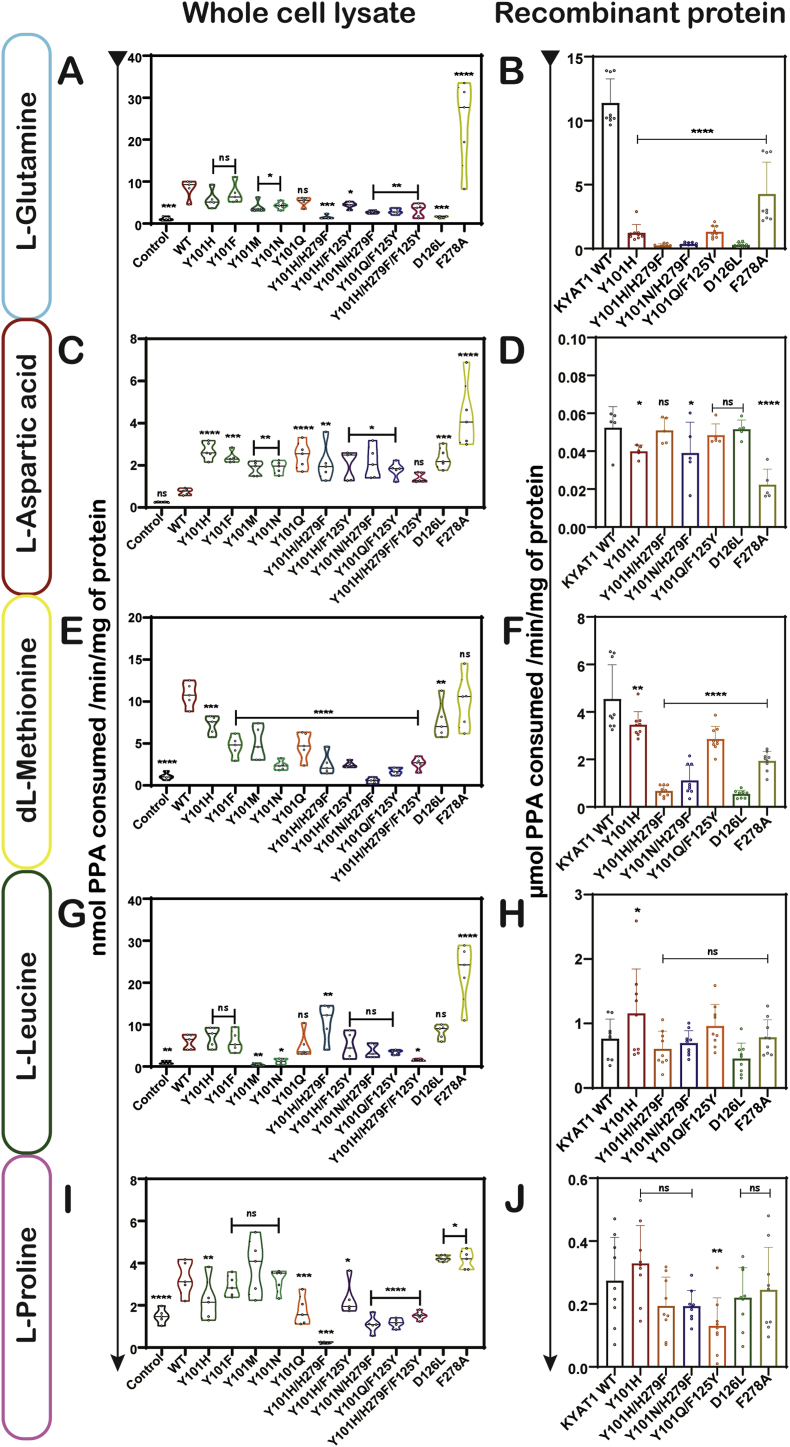
**Effect of hKYAT1 wild-type and mutation with the different amino acid substrates.** Transamination activity assays were done with substrates: L-Gln, L-Asn, dL-Met, L-Leu, and L-Pro (**A, C, E, G, I**) cell lysate, (**B, D, F, H, J**) recombinant protein. (**A-J**)The graph represents mean ± SD, statistical analysis performed with one-way ANOVA with 95 % confidence interval followed by Tukey's multiple comparison test (ns = not significant, *p < 0.05, **p < 0.01, ***p < 0.001, and ****p < 0.0001 compared with wild-type KYAT1) (n = 6).

Overall, amidic and polar substrates including L-Gln, L-Asp, L-Asn, and L-Cyss were metabolized less efficiently. However, the F278A mutant displayed again superior activity relative to wild-type, while mutants like Y101Q/F125Y and Y101H/H279F did not show any or very little effect. The enhanced activity of Y101H and Y101F toward L-Gln, L-Asn and L-Cyss suggests a broader catalytic flexibility (Fig. 7A–D, Supplementary Fig. 2E–H). Aliphatic substrates like dL-Met, L-Leu, L-Pro, L-Ala, and Gly were transaminated with low efficiency by all KYAT1 variants. F278A showed a modest increases in transamination activity over wild-type, while most Y101 mutants retained only baseline activity (Fig. 7E–J, Supplementary Fig. 2I–L). This suggests that the active site remains preferentially tuned for aromatic or bulky side chains, and that further engineering would be needed to improve reactivity with small aliphatic residues. Finally, SeMet, structurally analogous to MSC, was tested for both transamination and β-elimination. The Y101H, Y101F, Y101 N, Y101Q, F278A, and D126L mutants showed enhanced transamination activity toward SeMet relative to wild-type in cell lysates, suggesting that their engineered plasticity extends to other selenium compounds (Supplementary Fig. 3A–C). Kinetic analysis confirmed these trends, with Y101H and F278A mutants exhibiting broader substrate specificity and improved catalytic efficiency across most tested substrates (Table 1, Supplementary Fig. 3C). Furthermore, D126L mutant showed minimal β-elimination activity with SeMet, while F278A showed an enhanced activity in recombinant enzymes. A summary of the relative activity of wild-type and mutants like Y101H, Y101F, F278A and D126L across a panel of amino acid substrates is presented in Supplementary Table 2.

Altogether, these findings indicate that mutations in key substrate-interacting residues, particularly Y101, D126 and F278, can expand the catalytic repertoire of hKYAT1, offering new opportunities for rational enzyme design beyond MSC metabolism.

## 4 Discussion

This study demonstrates that structure-guided mutagenesis of human KYAT1 significantly alters its catalytic profile toward MSC, enhancing its therapeutic potential in liver cancer cells, and possibly other cancer models. By targeting residues at the substrate gate (Y101), the PLP-stabilizing region (D126), and the ligand-binding interface (F278), we engineered KYAT1 variants with enhanced transamination, β-elimination, or both, yielding increased MSC cytotoxicity and downstream epigenetic remodeling.

Among these, the Y101H mutant emerged as one of the most versatile variants, displaying superior transamination and β-elimination efficiency with MSC and aromatic substrates. hKYAT1's structural flexibility likely facilitates substrate accommodation and conformational dynamics, supported by kinetic data showing a >60-fold increase in catalytic efficiency over wild-type. Similarly, the F278A mutation, although catalytically moderate, exhibited broad substrate tolerance and dual-pathway activation, suggesting either an altered substrate orientation or a modified active site access due to the loss of aromatic stacking from the phenyl ring. In contrast, the D126L mutation abrogated transamination activity but retained and even enhanced β-elimination, reinforcing our understanding of the role of this residue as a structural anchor that stabilizes the positioning of the α1 helix, and controls entry-state transitions.

Our substrate screening further revealed that KYAT1 variants modulate activity not only toward MSC but also toward classical aromatic substrates (L-Phe, L-Trp, and L-Kyn). Notably, Y101H and D126L exhibited divergent effects on L-Kyn compared with L-Trp and L-Phe, underscoring how subtle structural changes can reconfigure the active-site environment to favor polar versus hydrophobic interactions. Consistent with this notion, our recent study showed that the H279F mutant enhanced transamination activity with both L-Phe and L-Trp [7]. In contrast, the Y101 N/H279F double mutant described here displayed diminished activity, consistent with a negative epistatic effect in which two individually favorable substitutions become deleterious in combination. The simultaneous loss of Y101 aromatic stacking (Y101 N) and H279 hydrogen-bonding capacity (H279F) likely disrupts substrate alignment for indole and aromatic substrates, explaining the reduced activity observed. Interestingly, the Y101H/H279F double mutant showed the opposite trend depending on the substrate: affinity was reduced for L-Trp but enhanced for L-Kyn. This contrasting outcome likely reflects how the two substitutions remodel the active site in a substrate-specific manner. While the loss of H279's polar side chain combined with altered positioning by Y101H destabilizes binding of the bulky indole moiety of Trp, the amide group of Kyn is better accommodated through interactions with the newly introduced Y101H imidazole. Such divergence underscores the highly context-dependent role of active-site residues and highlights how combined mutations can differentially tune substrate selectivity and catalytic plasticity.

These mechanistic distinctions are mirrored in cellular responses. Mutants with high β-elimination activity (Y101H, F278A, and D126L) induced strong caspase activation, cytochrome *c* release, and chromatin remodeling, classical hallmarks of mitochondrial apoptosis [20]. The complementary oxidative-stress assays collectively establish that KYAT1 activity dictates both the magnitude and localization of MSC-induced redox perturbations. The pronounced responses of D126L, characterized by elevated extracellular H_2_O_2_, lipid peroxidation, and mitochondrial superoxide, align with its selective enhancement of β-elimination and increased generation of methylselenol, a highly reactive metabolite capable of propagating oxidative cascades. In contrast, mutants including F278A, which maintain dual transamination and β-elimination activities, produced strong yet more balanced redox signatures, suggesting that the coexistence of both catalytic routes may buffer excessive ROS amplification. The convergence of GSH depletion, lipid oxidation, and mitochondrial dysfunction with HDAC downregulation and caspase activation supports a mechanistic framework in which selenium catabolism through engineered KYAT1 variants links redox stress to epigenetic and apoptotic signaling. These findings extend the biochemical observations to the cellular level and underscore how precise tuning of KYAT1 catalytic bias can modulate therapeutic redox pressure in cancer cells.

Moreover, increased histone H4 acetylation and reduced HDAC3/8 levels point to MSC-derived metabolites that affect epigenetic regulation [13,21]. Importantly, these effects were mutation-specific and not merely a function of protein expression level, underscoring the functional impact of KYAT1 engineering. The structural plasticity introduced by these KYAT1 mutations not only boosts MSC metabolism but also enables selective pathway preference, offering an innovative strategy to fine-tune the balance between transamination and β-elimination. The capacity to channel substrate flux into specific cytotoxic pathways makes the use of engineered hKYAT1 an attractive candidate for targeted cancer therapy. This concept parallels prodrug-enzyme therapy platforms like gene-directed enzyme prodrug therapy (GDEPT), but with the added advantage of dual-function catalysis and a redox-active substrate like MSC.

Interestingly, the differential modulation of HDACs and apoptotic regulators by various mutants suggests that MSC-derived metabolites like β-methylselenopyruvate and methylselenol exert epigenetic and mitochondrial stress in a mutation-dependent fashion. The increase in H4K16 acetylation and HDAC1/2 redistribution observed in Y101H- and D126L-expressing cells supports a model where hKYAT1 serves as both a catalytic and epigenetic control node under selenium treatment. Such dual functionality may be exploited for synthetic lethality-based strategies, especially in HDAC-addicted or apoptosis-resistant tumors. From a translational standpoint, further development of hKYAT1-based MSC therapies will require targeted delivery platforms to avoid systemic toxicity. Lipid nanoparticle (LNP) systems and affibody-decorated mRNA vectors have shown promise for selective enzyme expression in tumors [[22], [23], [24]]. Our previous work has demonstrated the utility of mRNA-based KYAT1 delivery in hepatocellular carcinoma models [12]. Combining this approach with catalytically enhanced hKYAT1 variants like Y101H, F278A, or D126L may achieve superior efficacy at lower MSC doses, improving therapeutic index.

**Supplementary Fig. 4:**
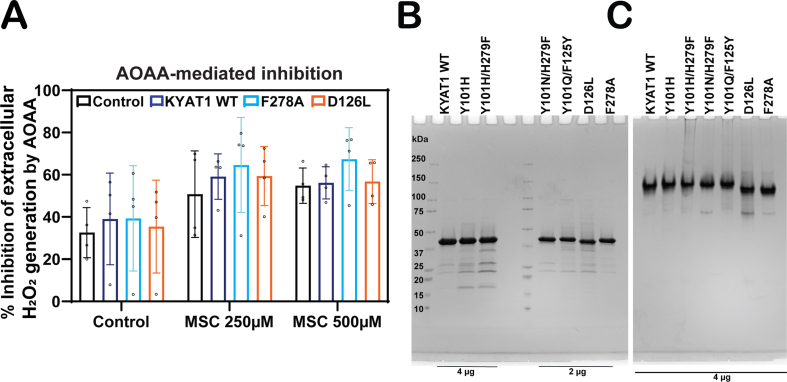
Aminooxyacetic acid-mediated inhibition of extracellular H_2_O_2_ and native characterization of recombinant KYAT1 proteins. **(A)** Effect of aminooxyacetic acid (AOAA, 1 mM) on MSC-induced extracellular H_2_O_2_ generation in Huh7 cells transfected with empty vector, KYAT1 wild-typr, or mutant constructs. Percentage inhibition was calculated as 100 x [1− (rate + AOAA / rate − AOAA)]. Data represent mean ± SD from n = 4 independent experiments. Statistical analysis was performed using one-way ANOVA (95 % confidence interval) followed by Dunnett's multiple comparison test (*ns* = not significant, *p < 0.05, **p < 0.01, ***p < 0.001, and ****p < 0.0001 compared with wild-type KYAT1). (**B**) SDS-PAGE and **(C)** Native PAGE analysis of purified recombinant KYAT1 wild-type and mutants protein, demonstrating identical molecular mass, charge distribution, hydrodynamic radius and native quaternary structure across variants.

Despite these advances, the study has limitations. First, although all recombinant hKYAT1 proteins were expressed under identical conditions and verified for integrity by SDS-PAGE and native gel electrophoresis (Supplementary Fig. 4B and 4C), additional biophysical analyses would further substantiate conformational stability and folding differences among the mutants. Structural validation via X-ray crystallography or computational modeling would solidify the proposed conformational mechanisms underlying mutant effects. Second, while liver cancer cells provide a useful model, future studies should expand to additional tumor types, including pancreatic or colorectal cancers, where MSC and its analogs have shown potential [25,26]. Finally, metabolomic profiling of MSC-derived intermediates could further clarify the link between enzyme kinetics and downstream signaling [27,28]. In conclusion, our findings demonstrate that rationally engineered hKYAT1 variants can reprogram selenium metabolism and trigger epigenetic and apoptotic changes in cancer cells. This work provides a blueprint for optimizing selenium-based therapeutics through enzyme-guided precision strategies.

## Funding

This study was supported by grants from Cancerfonden (23 2796 Pj 01H), Cancer och Allergifonden, Radiumhemmets forskningsfonder (231082), CIMED (FoUI-976014), Karolinska Institutet to M.B, and by grants from the Swedish Cancer Society (21 1605 Pj 01H and 24 3775 Pj 01H), the Cancer and Allergy Foundation (10399), the Swedish Research Council (2021-05061 and 2018-02874), and the King Gustaf V Jubileum Foundation (244092) to A.A.

## CRediT authorship contribution statement

**Arun Kumar Selvam:** Conceptualization, Data curation, Formal analysis, Investigation, Methodology, Project administration, Software, Supervision, Validation, Visualization, Writing – original draft, Writing – review & editing. **Renhua Sun:** Methodology, Writing – review & editing. **Tatiana Sandalova:** Conceptualization, Investigation, Project administration, Writing – original draft, Writing – review & editing. **Hugh Salter:** Conceptualization, Supervision, Writing – review & editing. **Adnane Achour:** Conceptualization, Funding acquisition, Project administration, Resources, Supervision, Writing – review & editing. **Mikael Björnstedt:** Conceptualization, Funding acquisition, Project administration, Supervision, Visualization, Writing – original draft, Writing – review & editing.

## Declaration of competing interest

M.B. is listed as an inventor in a patent application for i.v. Use of inorganic selenium in cancer patients and holds shares in SELEQ OY, a company involved in the development of Se-based formulations for prevention and treatment.

## Data Availability

Data will be made available on request.
