# Supplementary material for: Structural and functional modulation of human kynurenine aminotransferase I enhances selenium-driven redox metabolism for cancer therapy
Source: Redox Biol. 2025 Dec 9;89:103967. doi: 10.1016/j.redox.2025.103967 (PMC12808825; doi:10.1016/j.redox.2025.103967)
Supplement: Multimedia component 1 [file mmc1.docx]

Structural and functional modulation of human kynurenine aminotransferase I enhances selenium-driven redox metabolism for cancer therapy

Arun Kumar Selvam ^1^, Renhua Sun ^2^, Tatiana Sandalova ^2^, Hugh Salter ^1^, Adnane Achour ^2,^*, and Mikael Björnstedt ^1,^*

**Supplementary table 1:** List of antibodies

| Ab Nr | Ab name | Dilution | Diluted in | Company | Animal | Clonality |
| --- | --- | --- | --- | --- | --- | --- |
| #4370S | Phospho-p44/42 MAPK (Erk1/2)(Thr202/Tyr204) | 1:500 | 5%BSA | Cell signaling | Rabbit | monoclonal |
| #9662S | Caspase-3 | 1:1000 | 5%BSA | Cell signaling | Rabbit | monoclonal |
| #2920S | Akt (pan) | 1:1000 | 5%BSA | Cell signaling | Mouse | monoclonal |
| #13534S | Acetyl-Histone H4 (Lys16) | 1:100 | 5%BSA | Cell signaling | Rabbit | monoclonal |
| #2056S | PKCα Antibody | 1:500 | 5%BSA | Cell signaling | Rabbit | polyclonal |
| #8690S | p38 MAPK | 1:1000 | 5%BSA | Cell signaling | Rabbit | monoclonal |
| #9532S | PARP | 1:500 | 5%BSA | Cell signaling | Rabbit | monoclonal |
| #05-386 | Anti-Vinculin | 1:2000 | 5%BSA | MilliPore | Mouse | monoclonal |
| #14269S | Histone H3 | 1:2000 | 5%BSA | Cell signaling | Mouse | monoclonal |
| #61060 | Bcl-xL (54H6) | 1:1000 | 5%BSA | Cell signaling | Rabbit | monoclonal |
| #89477 | Bax | 1:1000 | 5%BSA | Cell Signaling | Mouse | monoclonal |
| sc-7907 | PCNA | 1:1000 | 5%BSA | Santa Cruz Biotechnology | Rabbit | polyclonal |
| #15071 | Bcl-2 | 1:1000 | 5%BSA | Cell Signaling | Mouse | monoclonal |
| K333-11-30 | HDAC1 | 1:1000 | 5%BSA | BioVision | Rabbit | polyclonal |
| K333-11-30 | HDAC2 | 1:1000 | 5%BSA | BioVision | Rabbit | polyclonal |
| #3949 | HDAC3 | 1:1000 | 5%BSA | Cell Signaling | Mouse | monoclonal |
| #66042 | HDAC8 | 1:1000 | 5%BSA | Cell Signaling | Rabbit | monoclonal |
| #12165S | HSP60 (D6F1) | 1:2000 | 5%BSA | Cell Signaling | Rabbit | monoclonal |
| #11940S | Cytochrome c (D18C7) | 1:1000 | 5%BSA | Cell Signaling | Rabbit | monoclonal |
| #94296S | Mcl-1 (D2W9E) | 1:1000 | 5%BSA | Cell Signaling | Rabbit | monoclonal |
| #2224 | Caspase 2 (C2) | 1:1000 | 5%BSA | Cell Signaling | Mouse | monoclonal |
| #20750 | Cleaved Caspase-9 (Asp315) | 1:1000 | 5%BSA | Cell Signaling | Rabbit | monoclonal |
| #9461 | Cleaved Caspase-7 (Asp198) | 1:1000 | 5%BSA | Cell Signaling | Rabbit | monoclonal |
| #4696 | p44/42 MAPK (Erk1/2) | 1:1000 | 5%BSA | Cell signaling | Mouse | monoclonal |

**Supplementary table 2: KYAT1 Substrate Specificity Summary**

Relative transamination activity of wild-type and mutant KYAT1 enzymes across a panel of amino acid substrates. Ratings: ++++ = Strongly enhanced, +++ = Moderately enhanced, ++ = Wild-type level, + = Weak activity, - = No detectable activity.

| Substrate | WT | Y101H | F278A | D126L | Y101F |
| --- | --- | --- | --- | --- | --- |
| MSC | ++ | +++ | ++++ | + | +++ |
| SeMet | + | ++ | ++ | ++ | ++ |
| L-Phe | ++ | +++ | ++++ | +++ | ++ |
| L-Trp | + | ++ | ++++ | +++ | +++ |
| DL-Tyr | ++ | + | +++ | + | +++ |
| L-His | ++++ | ++ | +++ | + | ++ |
| L-Kyn | + | + | +++ | ++++ | ++ |
| L-Gln | + | + | ++ | - | + |
| L-Cys | + | + | ++++ | ++++ | ++ |
| L-Ala | + | + | - | + | ++ |
| Gly | + | - | + | + | - |
| L-Leu | + | + | ++ | + | + |
| L-Asn | + | + | ++ | + | + |
| L-Asp | + | ++ | +++ | ++ | ++ |
| Pro | ++ | + | ++ | ++ | + |
| DL-Met | +++ | ++ | ++ | + | + |
